# Dissemination of *Mycobacterium abscessus* via global transmission networks

**DOI:** 10.1038/s41564-021-00963-3

**Published:** 2021-09-20

**Authors:** Christopher Ruis, Josephine M. Bryant, Scott C. Bell, Rachel Thomson, Rebecca M. Davidson, Nabeeh A. Hasan, Jakko van Ingen, Michael Strong, R. Andres Floto, Julian Parkhill

**Affiliations:** 1 Molecular Immunity Unit, University of Cambridge Department of Medicine, MRC-Laboratory of Molecular Biology, Cambridge, UK; 2 Department of Veterinary Medicine, University of Cambridge, Cambridge, UK; 3 Children’s Health Research Institute, The University of Queensland, Brisbane, Australia; 4 Thoracic Medicine, The Prince Charles Hospital, Brisbane, Australia; 5 Gallipoli Medical Research Institute, The University of Queensland, Brisbane, Australia; 6 Center for Genes, Environment and Health, National Jewish Health, Denver, CO USA; 7 Center for Infectious Diseases, Department of Medical Microbiology, Radboud University Medical Center, Nijmegen, Netherlands; 8 Cambridge Centre for Lung Infection, Papworth Hospital, Cambridge, UK; 9 Cambridge Centre for AI in Medicine, University of Cambridge, Cambridge, UK

**Keywords:** Bacterial genetics, Pathogens, Genetic variation

## Abstract

*Mycobacterium abscessus*, a multidrug-resistant nontuberculous mycobacterium, has emerged as a major pathogen affecting people with cystic fibrosis (CF). Although originally thought to be acquired independently from the environment, most individuals are infected with one of several dominant circulating clones (DCCs), indicating the presence of global transmission networks of *M. abscessus*. How and when these clones emerged and spread globally is unclear. Here, we use evolutionary analyses of isolates from individuals both with and without CF to reconstruct the population history, spatiotemporal spread and recent transmission networks of the DCCs. We demonstrate synchronous expansion of six unrelated DCCs in the 1960s, a period associated with major changes in CF care and survival. Each of these clones has spread globally as a result of rare intercontinental transmission events. We show that the DCCs, but not environmentally acquired isolates, exhibit a specific smoking-associated mutational signature and that current transmission networks include individuals both with and without CF. We therefore propose that the DCCs initially emerged in non-CF populations but were then amplified and spread through the CF community. While individuals with CF are probably the most permissive host, non-CF individuals continue to play a key role in transmission networks and may facilitate long-distance transmission.

## Abstract (web summary)

In this Article, the authors perform evolutionary analyses of *M. abscessus* clinical isolates and report the emergence of dominant circulating clones (DCCs) in non-cystic fibrosis (CF) individuals followed by amplification in the CF community.

## Main

*Mycobacterium abscessus* is a multidrug-resistant species of nontuberculous mycobacteria (NTM) that has recently emerged as a major threat to individuals with CF, with increasing rates of infection seen in CF cohorts around the world^1^. This rapidly growing NTM is divided into three subspecies: *M. abscessus* subspecies *abscessus* (*M. a. abscessus*), *M. a. massiliense* and *M. a. bolletii*^2^. Infections with *M. abscessus* lead to accelerated inflammatory lung damage^3^, are often difficult or impossible to treat despite prolonged courses of combination antibiotics^4–6^ and may prevent safe lung transplantation^1,7^.

Although *M. abscessus* was originally thought to only be independently acquired from the environment, we^8^ and others^9^ have shown that individuals with CF can become infected through hospital-based person-to-person transmission (probably through the generation of long-lived infectious aerosols or via fomite spread^2^). Indeed, large scale whole genome sequencing of *M. abscessus* isolates from CF centres around the world has revealed that most individuals with CF are infected with one of three highly prevalent globally dispersed clones, referred to as DCCs 1–3 (ref. ^2^). Assigning the DCC classification onto whole genome sequences from other studies indicates that the DCCs can also infect non-CF individuals^10,11^. DCC isolates are associated with worse clinical outcomes, have greater antibiotic resistance and are more virulent in in vitro and in vivo infection models^2^, suggesting that multiple rounds of within-host evolution have promoted increased pathogenic potential.

The high levels of genetic relatedness within the DCCs suggest that they have emerged recently and have rapidly spread within and between countries, as well as across continents^2,10,12,13^. There are many examples of isolates from individuals in different CF centres or in different countries whose sequences differ by fewer mutations than have been seen in a single individual during chronic infection^2,14^, suggesting individuals are linked by recent and widespread transmission networks. However, despite multiple studies combining whole genome sequencing with epidemiological data^2,8,9,13–18^, the relative importance of different routes of acquisition remains unclear. In addition, the mechanism of transcontinental spread of DCCs^6^ (given the lack of movement of CF individuals) is unknown. Several studies have found near-identical isolates in CF individuals with no obvious epidemiological links^15–18^, suggesting that transmission chains include additional unknown links, potentially implicating environmental or human intermediates.

To understand how the *M. abscessus* DCCs emerged and the routes through which they have spread globally, we have applied evolutionary phylogenetic analyses to whole genome sequences of clinical isolates from 1,178 individuals on five continents. We show that the DCCs expanded synchronously around the time of increases in CF life expectancy, spread globally (particularly from Europe to North America) and contain a mutagenic signature of exposure to smoking-related mutagens during their expansion, implicating smokers and more generally non-CF individuals, as an important second human niche for *M. abscessus*.

## Results

### Synchronous clonal expansions coincident with the emergence of the CF lung niche

**Fig. 1:**
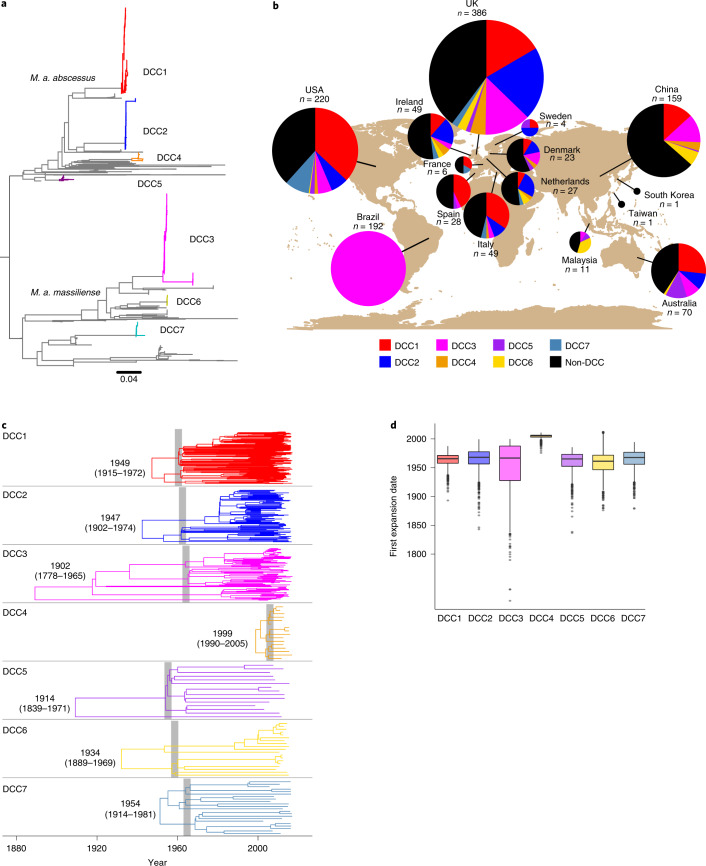
Emergence of *M. abscessus* DCCs. **a**, The phylogenetic placement of the DCCs in the *M. abscessus* species phylogenetic tree. Several non-DCC clades have been collapsed for clarity; the full tree is presented in Extended Data Fig. 1. **b**, The proportion of individuals from each location infected with each DCC. All samples from Brazil are from a surgical outbreak caused by a single lineage^30^. **c**, Temporal maximum clade credibility phylogenetic tree of each DCC. DCC most recent common ancestor dates are shown as median with 95% HPD in parentheses. Shaded areas show the first period of rapid divergence in each DCC. **d**, Expansion date distributions for each DCC as calculated from the complete posterior distribution of trees (number of samples from the posterior distribution: DCC1 = 2,255, DCC2 = 2,255, DCC3 = 2,255, DCC4 = 2,699, DCC5 = 2,702, DCC6 = 2,613, DCC7 = 2,703). Boxplot centre lines show median value; upper and lower bounds show the 25th and 75th quantile, respectively; upper and lower whiskers show the largest and smallest values within 1.5 times the interquartile range above the 75th percentile and below the 25th percentile, respectively; and points show samples outside the whisker range. Source data

**Extended Data Fig. 1:**
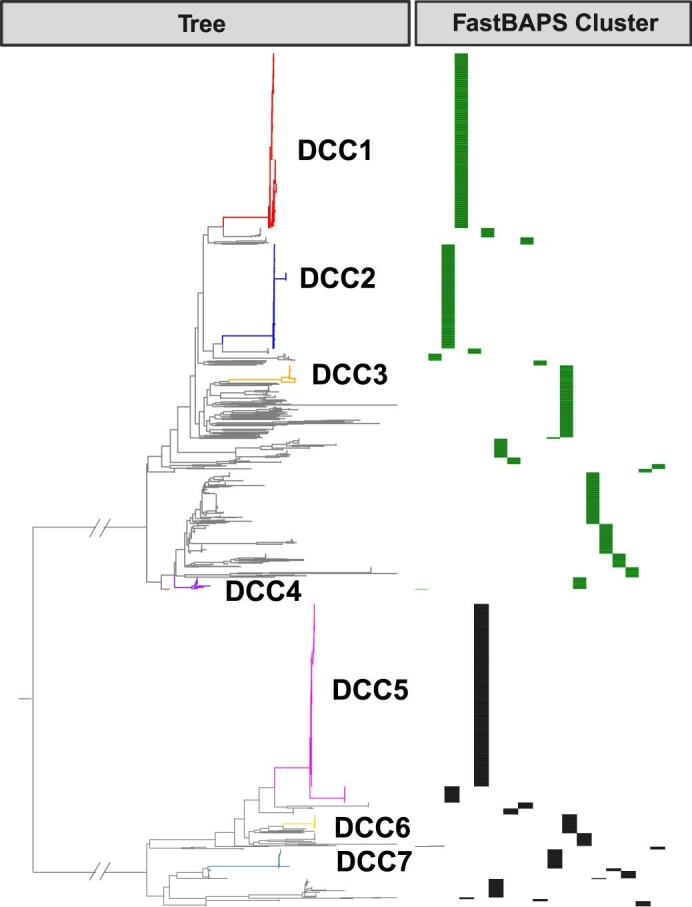
Population structure of *M. abscessus*. A phylogenetic tree was reconstructed containing all isolates from all individuals (n = 1335 for *M. abscessus subsp abscessus* (*M. a. abscessus)*, n = 710 for *M. a. massiliense*). As Fig. 1a but no clades were collapsed in this tree. The sequences that cluster within each DCC are labelled. FastBAPS clusters are plotted next to the tree for *M. a. abscessus* (green) which contains 19 FastBAPS clusters and *M. a. massiliense* (black) which contains 17 FastBAPS clusters. Source data

We first reconstructed the phylogenetic relationships between 2,045 *M. abscessus* whole genome sequences from 1,178 individuals to identify recently emerged clones whose ancestry could be dated. We identified seven clonal clusters, including the three previously identified DCCs^2^, that contained highly related isolates from at least 20 individuals (Fig. 1a and Extended Data Fig. 1) and were found on multiple continents (Fig. 1b and Supplementary Table 1). We classified these clones as DCC1–7.

Bayesian temporal reconstruction^19^ indicated that each of the DCCs emerged relatively recently, with their most recent common ancestors dating between 1902 and 1999 (Fig. 1c). The dated phylogenetic tree of each DCC is characterized by a section with multiple concurrent short branches, indicating a historical period of rapid expansion from a small number of lineages into multiple independent transmission chains that have persisted to the present day (Fig. 1c). In support of this conclusion, we found strong statistical evidence (posterior probability of one for each DCC, Methods) that each DCC had undergone at least one historical population expansion, the first of which occurred for six of the seven DCCs in the 1960s and for DCC4 much later (Fig. 1d).

**Extended Data Fig. 2:**
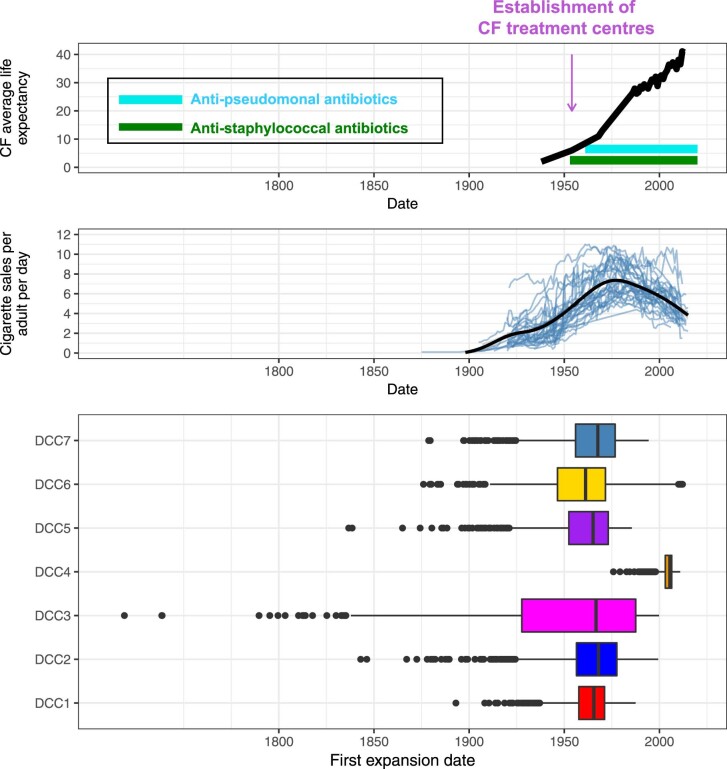
Drivers of the concurrent DCC expansion. The bottom panel shows the expansion date distribution for each of the DCCs, as in Fig. 1d (number of samples from the posterior distribution: DCC1 = 2255, DCC2 = 2255, DCC3 = 2255, DCC4 = 2699, DCC5 = 2702, DCC6 = 2613, DCC7 = 2703). Boxplot centre lines show median value, upper and lower bounds show the 25^th^ and 75^th^ quantile, respectively, upper and lower whiskers show the largest and smallest values within 1.5 times the interquartile range above the 75^th^ percentile and below the 25^th^ percentile, respectively, and points show samples outside of the whisker range. The top panel shows factors within the CF population that likely drove DCC expansion. The middle panel shows the trend in cigarette sales in developed countries; each blue line is the trend in a single country and the black line is the average trend across countries combined. Source data

We proposed that the expansion in DCCs could have been driven by changes within the CF population since *M. abscessus* has become a main cause of lung infection in individuals with CF^1^, can transmit onwards from CF individuals^2,8^ and is more prevalent in this cohort than in other patient groups^20^. We found that the DCC expansion occurred shortly after life expectancy of CF individuals began to increase from infancy to over 10 years of age in the 1950s and 1960s (Extended Data Fig. 2)^21,22^. This period also saw the establishment of CF treatment centres and the widespread use of antipseudomonal and antistaphylococcal antibiotics^21^ (to which *M. abscessus* is intrinsically resistant) (Extended Data Fig. 2), suggesting that the synchronous expansion of DCCs in the 1960s may have been driven both by increases in the number and density of susceptible individuals (promoting person-to-person transmission^2,8^) and also the creation of a more permissive lung niche for *M. abscessus*, through suppression of other lung pathogens^1,23^. The more recent expansion of DCC4 in the mid-2000s (Fig. 1d) suggests that suitable conditions for the emergence of new clones have persisted within present day patient cohorts.

### Rare intercontinental transmission events drive global transmission networks

**Fig. 2:**
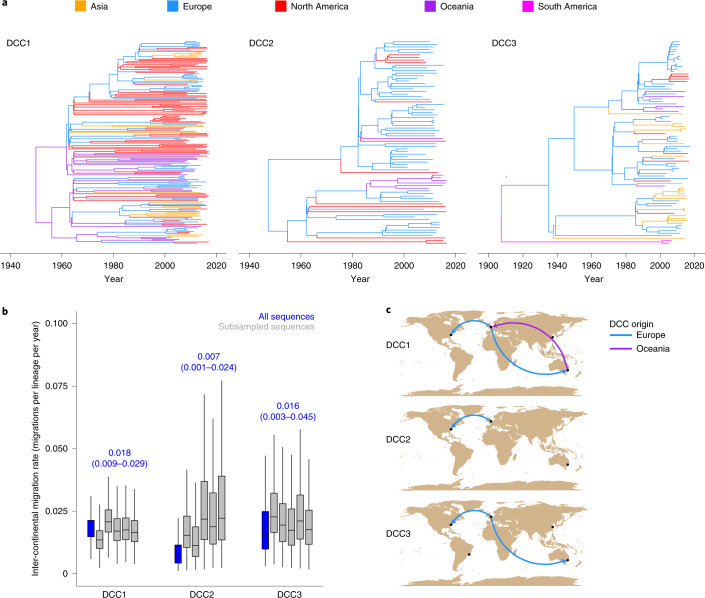
Global migration network of the DCCs. **a**, Phylogeographic trees for DCCs 1–3. Each branch is coloured by continent. **b**, Intercontinental migration rates for each DCC for the complete dataset and five random subsamples (Methods, number of samples from the posterior distribution: DCC1 complete dataset of 3,157, all DCC1 subsampled datasets, DCC2 subsampled datasets 1, 3 and 5, all DCC3 subsampled datasets of 2,703 each; DCC2 complete dataset, DCC2 subsampled datasets 2 and 4, DCC3 complete dataset of 2,256 each). Boxplot centre lines show median value; upper and lower bounds show the 25th and 75th quantile, respectively; upper and lower whiskers show the largest and smallest values within 1.5 times the interquartile range above the 75th percentile and below the 25th percentile, respectively; and points show samples outside the whisker range. The median and 95% HPD of the complete DCC datasets are shown above the bars of the corresponding DCC. An intercontinental migration rate of 0.02 migrations per lineage per year corresponds to one in 50 lineages moving continent in a year. **c**, Intercontinental migration pathways for each DCC. Arrows show the direction of supported migration from source continent to recipient continent. Arrows are coloured by originating continent. Source data

**Extended Data Fig. 3:**
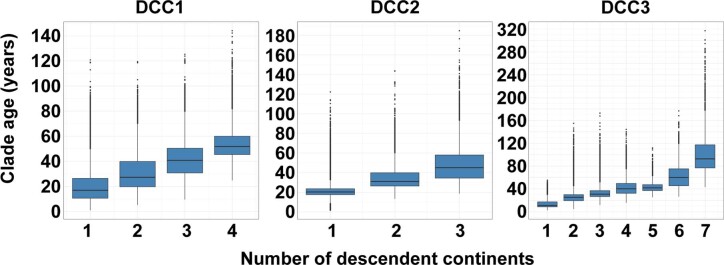
Older phylogenetic clades contain sequences from more continents than more recent phylogenetic clades. The age and number of continents descendent sequences were sampled from was calculated for all clades across the posterior distribution of phylogenetic trees (number of clades: DCC1 = 539847, DCC2 = 173635, DCC3 = 182655). Boxplots depict all clades from all trees. The distribution of clades ages is significantly different for all pairs of continent numbers for all DCCs (Bonferroni corrected two sample two-sided Kolmogorov-Smirnov test, p < 0.001). Source data

We next investigated the global transmission network of *M. abscessus* by reconstructing the broad-scale geographical distribution of the three most prevalent clones, DCCs 1–3. In each case, isolates from different continents are interspersed within the phylogenetic tree (Fig. 2a), demonstrating historical intercontinental transmission events that are relatively uncommon, with approximately one out of every 50 lineages moving between continents each year (Fig. 2b). Furthermore, we found a significant correlation between the age of a clade and the number of continents it has been detected in, with most newly emerged clades only present in one continent (Extended Data Fig. 3). However, despite these low overall rates of intercontinental transmission, we found strong evidence for migration of *M. abscessus* between specific pairs of continents and, in particular, of migration of all three main DCCs from Europe to North America (Fig. 2c), potentially driven by the high density of CF individuals in Europe^24,25^.

### Mutational spectrum analysis implicates smokers in DCC transmission networks

While our results indicate a major role for individuals with CF in the expansion of DCCs, it is clear from our temporal reconstruction that several of the DCCs emerged within the human population before CF survival increased beyond early childhood (Fig. 1c), suggesting an alternative pre-existing niche. Similarly, the transcontinental spread of DCCs is difficult to explain without an alternative human vector for transmission, given the historically limited travel of CF individuals.

We therefore proposed that individuals who smoke, given their recognized predisposition to mycobacterial infection^26–29^, might have provided the early niche for DCC emergence and facilitated their global dissemination. To test this hypothesis, we performed a mutational spectrum analysis of the *M. abscessus* genomes. The mutational spectrum is the pattern of different types of DNA mutation arising as a consequence of the action of various mutagenic processes and DNA repair mechanisms over time^30,31^. Since bacteria are likely to be exposed to different mutagens during pulmonary infection than when in the environment, we reasoned that we could use mutational spectrum analysis to define the historical exposure of DCCs to these different niches.

**Fig. 3:**
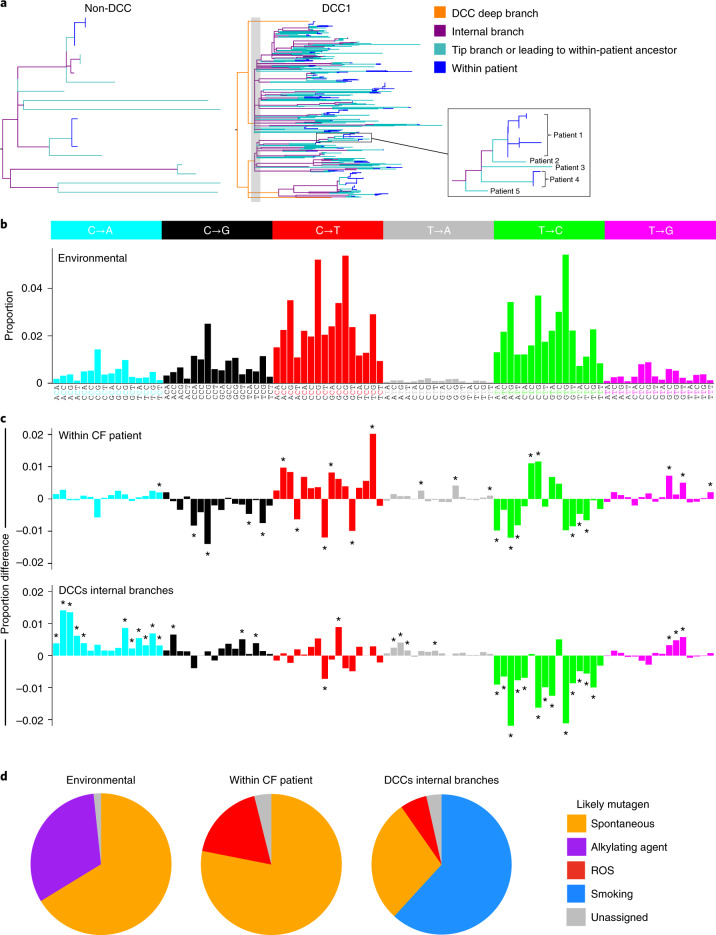
DCCs exhibit a smoking-related mutational spectrum. **a**, Branch classification for each branch type. The left-hand tree illustrates the categorization of non-DCC branches into: within patient, which occur downstream of a node in the tree where all of the descendent sequences are from the same patient; tip branches and branches leading to within-patient clade ancestors where some of the evolution may have occurred within a patient; and internal branches where all the change is likely to have occurred in the environment. The right-hand tree illustrates the additional categorization of DCC branches into deep branches that precede the first population expansion. Note that in DCCs, all branch categories are likely to be within patients, not the environment. **b**, Inferred environmental mutational spectrum of *M. abscessus* as calculated from the internal branches of the non-DCCs. The mutational spectrum consists of six different mutations, shown in different colours, each in 16 different nucleotide contexts^25^. The bars show the proportion of the total mutations of that type in that context along the non-DCC internal branches. **c**, Mutational spectra attributable to mutations occurring (top) within patients with CF (on the basis of longitudinal isolates collected from infected individuals), and (bottom) during expansion and dissemination of the DCCs (on the basis of analysis of the internal phylogenetic branches of the DCCs excluding the deep branches, that is, after the population expansion). Results are shown as the difference from the environmental spectrum. Asterisks indicate significantly different proportions relative to the environmental mutational spectrum, calculated through permutation analysis. **d**, Deconvolution analysis reveals relative contributions of specific mutational processes to each spectrum: unassigned (grey), spontaneous (yellow), alkylating agent–associated (purple), ROS-associated (red) and smoking-related (blue). Source data

We first calculated the spectrum of mutations in *M. abscessus* attributable to the environment, by analysing mutations occurring along the internal phylogenetic branches of the non-DCC clades, which constitute long periods of predominantly or exclusively environmental replication (Fig. 3a,b). We were then able to define the specific mutational profile arising only during chronic infection of CF individuals (by examining within-patient longitudinal samples), and the profile generated during the expansion and dissemination of DCCs (by looking at the internal branches of the DCC phylogenies), which were both notably different from the spectrum attributable to the environment and from each other (Fig. 3c).

Decomposition analysis^32^ (Fig. 3d) suggests that the environmental mutational spectrum consists of contributions from spontaneous mutation events (arising from cytosine deamination and inefficient homologous recombination repair) and mutations caused by alkylating agents, some of which have previously been reported to be present in soil^33,34^. In contrast, the within-CF patient spectrum has contributions from mutations associated with exposure to reactive oxygen species (ROS)^30^, thought to be generated at high levels within the CF lung^35^.

We found that the DCC-specific spectrum, in addition to having some contribution from ROS-related mutagenesis, was dominated by a general (context-independent) elevation in C-to-A/G-to-T mutations, a pattern seen in lung cancers from smokers^36^ and in human cells exposed to tobacco smoke in vitro^30^ (Fig. 3d). We can therefore infer that the DCC expansion and transmission networks involve smokers as well as individuals with CF.

### Individuals with CF and without CF (non-CF) are involved in DCC transmission networks

**Fig. 4:**
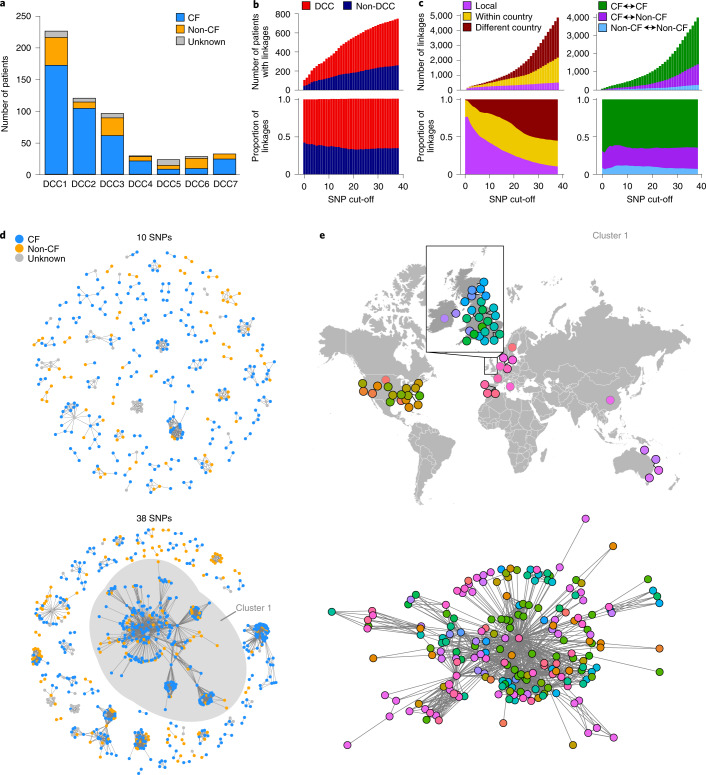
Transmission network analyses. **a**, Number of isolates in each DCC from patients with CF and without (non-CF). **b**, Number and proportion of patients infected with DCCs and non-DCC strains showing genetic links at various SNP cut-offs (*y* axis). **c**, Number and proportion of linkages of different geographical distances at various SNP cut-offs, and those that are between and within patients with and without CF. **d**, Network diagrams at 10 and 38 SNP cut-offs showing transmission clusters coloured by patients with and without CF. **e**, Detail of the largest cluster in the 38-SNP network showing the geographical location of patients and the network diagram coloured by geographical location. Nodes with black and grey outlines indicate city- and country-level information about isolate location, respectively. Source data

**Extended Data Fig. 4:**
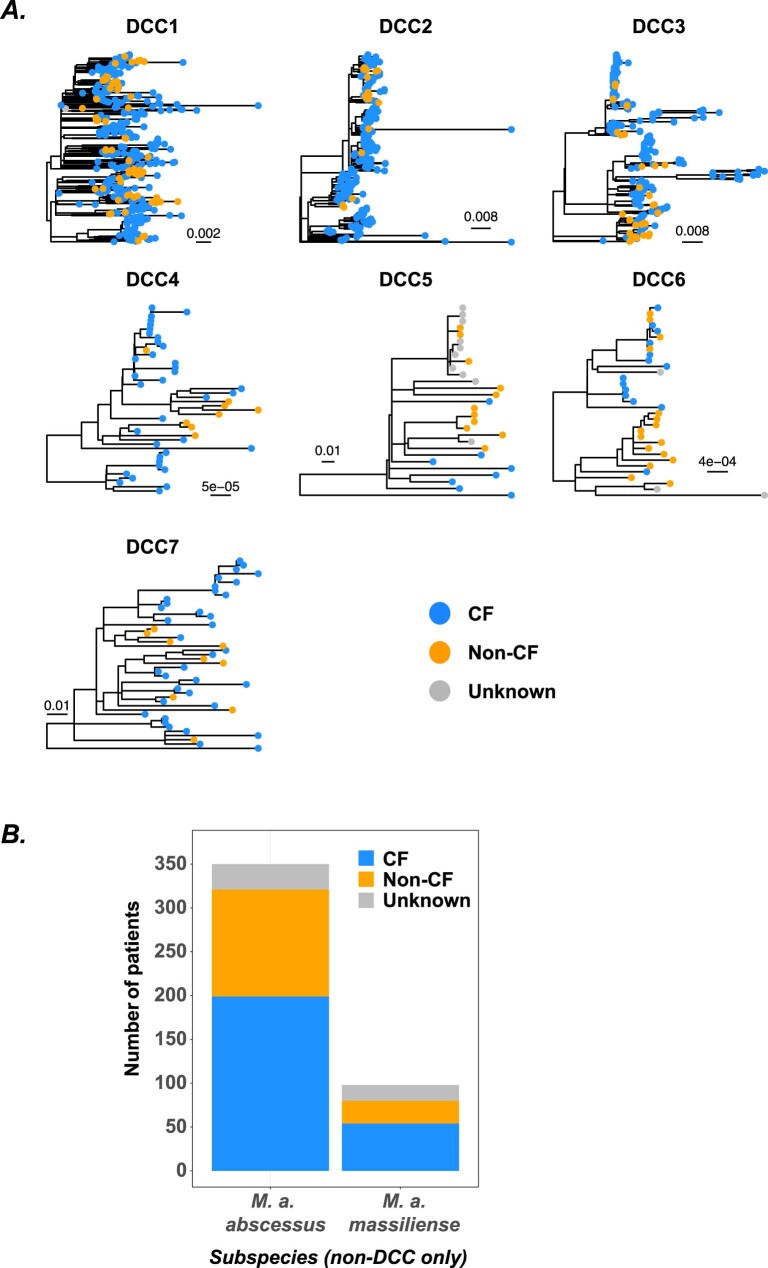
CF and non-CF patient isolates are intermingled in DCC phylogenetic trees and infected with non-DCCs. (**a**) Maximum likelihood phylogenetic trees are shown for each DCC with tips coloured by CF status. The scale bars show expected nucleotide substitutions per variable genome site. (**b**) All non-DCC isolates from each subspecies are combined. Source data

**Extended Data Fig. 5:**
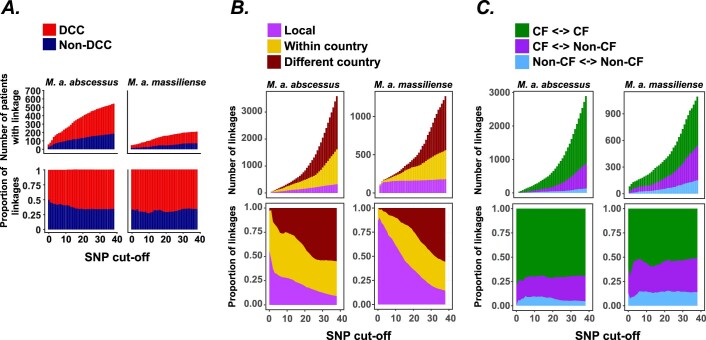
Transmission summaries divided into *M. a. abscessus and M. a. massiliense*. Pairs of individuals are classified as linked at a given SNP cut-off if their closest related isolates differ by fewer than that number of SNPs. (**a)** Linkages at each SNP cut-off split by subspecies. **(b)** Linkages were classified as local if the individuals are from the same hospital, CF center, city or state. Within country linkages are pairs of individual within the same country that do not meet the criteria for local. **(c)** Linkages were classified based on the CF status of the two individuals. Source data

**Extended Data Fig. 6:**
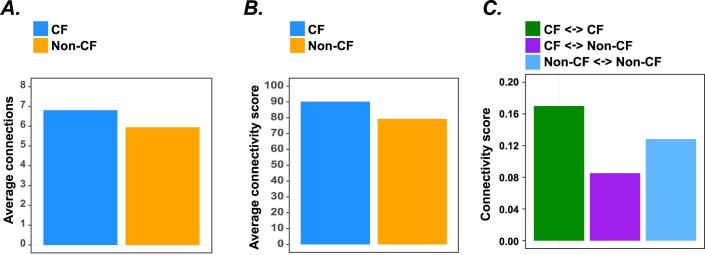
CF and non-CF individuals are well connected within the transmission network. Network connectivity scores were calculated from the transmission calculated at a cutoff of 38 SNPs presented in Fig. 4D. **(a)** The number of connections exhibited by each individual in the network was calculated and split into CF and non-CF individuals. The mean number of connections exhibited by an individual with the corresponding CF status is plotted. **(b)** Each connection in the transmission network was given a weighting based on the minimum SNP distance between isolates from the corresponding individuals, calculated as ‘39 – minimum SNP distance’. Therefore linkages representing fewer SNPs are given a higher weighting, the maximum weighting is 38 if isolates from two individuals differ by 0 SNPs and linkages at 38 SNPs have a weighting of 1. The weighted linkages of all individuals with the corresponding CF status were summed and divided by the number of individuals with that status. **(c)** Connections in the transmission network were divided into three categories based on the CF status of the two linked individuals: CF < - >CF, CF < - >non-CF, non-CF < - >non-CF. Linkages were weighted as in **(b)**. All weighted linkages within each type of linkage were summed and divided by the total number of potential linkages between individuals with the corresponding CF status. Source data

Given the mutational evidence of involvement of smokers in the initial emergence and continued transmission of the DCCs, we sought to quantify whether current transmission networks included both those with and without CF, or whether they form discrete transmission chains. We found that isolates from the lungs of individuals with and without CF could both be found in all seven DCCs (Fig. 4a, Extended Data Fig. 4a and Supplementary Table 2) and in non-DCCs (Extended Data Fig. 4b), showing that all of the emergent clones are capable of infecting members of either group. We then linked individuals on the basis of the genetic distance of their isolates in terms of single nucleotide polymorphisms (SNPs), using a range of linkage cut-offs that capture possible person-to-person transmission^2^. This demonstrated many close acquisition linkages between individuals within both DCC and non-DCC clades (Fig. 4b and Extended Data Fig. 5), showing that *M. abscessus* transmission is not limited to just the DCCs and raising the possibility of the emergence or discovery of more circulating clones in the future. We showed that local linkages dominate at smaller genetic distances, with national and then international linkages becoming more frequent at higher SNP distances (Fig. 4c and Extended Data Fig. 5); the expected signature of geographical spread of transmissible clones. We were also able to show that, at all genetic distances, linkages could be identified between individuals with CF, between individuals with and without CF and directly between individuals without CF (Fig. 4c and Extended Data Fig. 5) (although the relative proportions of these will be confounded by the different levels of sampling in the two groups). To confirm this apparent linkage between individuals with and without CF, we reconstructed transmission networks on the basis of SNP cut-offs of 10 and 38 SNPs (representing likely and possible transmission based on within-host diversity^2^, Fig. 4d). At both cut-off levels, transmission networks exist that include both individuals with and without CF, with each group exhibiting strong connectivity within the network (Extended Data Fig. 6). We further investigated the largest network (which consists of isolates from DCC1 from both individuals with and without CF, Fig. 4e) and found that this network had a global reach, with multiple international and national connections evident.

## Discussion

Halting the growing rate of infections with *M. abscessus* will require a comprehensive understanding of how individuals acquire these infections and how the bacteria spread at local and global scales. We have applied evolutionary analyses of whole genome sequences to reconstruct the population history, spatiotemporal spread and mutational history of *M. abscessus*. Our analysis demonstrates a key role for person-to-person transmission in *M. abscessus* epidemiology. The synchronized expansion of six of the seven DCCs (apparent despite their different sample numbers, sample density and tree depths) was tightly associated with a period of multiple changes in the CF population (Fig. 1d and Extended Data Fig. 2), indicating that the expansion was probably driven by the establishment of person-to-person transmission chains on the emergence of the CF lung niche. It is possible that these expansions may have additionally been promoted by increasing tobacco smoking rates in developed countries seen from the early 1900s to a peak around 1980 (Extended Data Fig. 2).

The rapid diversification in the DCC trees in the 1960s (Fig. 1c) can only be explained by multiple independent transmission chains that were established at this time, have persisted to the present day, and which involve both CF and non-CF individuals (Fig. 4). The age of these clones, the large number of individuals linked at very small SNP distances (Fig. 4), the high frequency of long range national and international linkages, and the correlation between genetic and geographical distance (Fig. 4c) are all patterns expected of a human lung pathogen and effectively rule out the hypothesis that a broadly spread genetically homogeneous environmental clone can explain the infection of CF individuals with related isolates^18^. While the main niche for the DCCs appears to be within human lungs, transmission is likely to be indirect and involve environmental and fomite intermediates^2,8^, which may lead to extrapulmonary DCC infections.

The smoking-related mutational signature that we identified within the DCCs (Fig. 3c,d) occurred along the internal phylogenetic branches within these clones, indicating that smokers have contributed to the expansion and continued transmission chains of *M. abscessus* DCCs. Smokers exhibit increased susceptibility to many infections, including NTMs^26,27,29,37,38^ and *Mycobacterium tuberculosis*^28^, due to structural lung changes and impaired immune responses^39–41^. Correspondingly, previous studies have frequently identified *M. abscessus* in smokers^6,29,38^. It therefore seems likely that tobacco smoke contributes to *M. abscessus* mutational burden, although overall substitution rates may be similar across different patient groups due to the action of other mutagens, such as high ROS levels in CF individuals^35^.

Our data therefore support a model where individuals with CF are the most permissive host for *M. abscessus* infection (as rapid expansion only occurred when this niche became available) but where smokers and other individuals without CF can act as an inefficient reservoir for infection and long-distance transmission. This supports recent evidence that emerging *M. abscessus* lineages are evolving to become human adapted^42^. Strong evidence of *M. abscessus* transmission from individuals with CF^2,8^ and higher rates of infection in this group compared with other susceptible groups^20^ support the importance of individuals with CF as key hosts for *M. abscessus*. Involvement of individuals without CF in transmission chains (Fig. 4) efficiently explains two previously puzzling observations; the missing epidemiological links between individuals with CF infected with highly genetically similar isolates identified in previous studies^15–17^, and the evidence for international transmission when individuals with CF have historically had limited intercontinental travel. Furthermore, while infections do occur within hospitals^8,9^, our data indicate that many transmission events occur in other settings. Future studies incorporating a greater number of isolates from individuals without CF will be needed to determine the relative transmission rates between the different groups.

The population expansion that we describe in the DCCs only represents the initial expansion of these clones and epidemiological data indicate that the DCCs have continued to increase in prevalence to the present day^1^. While additional mechanisms have probably contributed, our data indicate that increasing person-to-person transmission is the key driver of the increased prevalence of *M. abscessus*. Our previous work^2^ demonstrated phenotypic differences between clustered and unclustered *M. abscessus* isolates and comparison with the analysis here showed that that work included isolates from DCCs 1, 2, 3 and also from the newly identified 4 and 6 (but not DCCs 5 or 7). This shows that phenotypes associated with the clustered isolates are not limited to DCCs 1–3. However, further phenotyping comparing DCCs will be useful in future work. The seven DCCs are currently the only clones within the species whose expansion has been detected in our dataset. The more recent expansion of DCC4 (Fig. 1d) potentially occurred when this clone entered the population or had the opportunity to establish onward transmission. This coupled with the evidence for linkage between individuals carrying non-DCC strains (Fig. 4b), suggests that, as a species, *M. abscessus* is capable of human infection and transmission and that additional clones will continue to expand in the future when they have the opportunity to do so. Phylogeographic reconstruction (Fig. 2a) did not identify the regions within which early circulation occurred and it is therefore difficult to forecast where additional clones may expand in the future. However, our data indicate that it is reasonable to expect the early stages of expansion to occur within CF populations. Continued sequencing of clinical isolates and comparison with global sequences will be important to detect any additional clones that emerge in the future.

Further studies to understand how individuals without CF might transmit *M. abscessus* (both to individuals with and without CF) are vital and may provide information on how to reduce transmission risk. However, from previous studies^2,8,9^ it is reasonable to expect transmission to be indirect through the environment, and the role of individuals without CF in contaminating such environments requires particular attention. Comprehensive surveillance of potential environmental intermediates and isolates from individuals with and without CF may better define these transmission pathways.

In conclusion, reconstruction of the evolutionary history of the *M. abscessus* DCCs supports early emergence into non-CF populations, including smokers, followed by expansion due to person-to-person transmission that began with the emergence of the CF lung niche and has continued with contributions from individuals with and without CF. It also suggests that long-distance transmission may be due to low-level infection of individuals without CF. Our study demonstrates the use of combining whole genome sequencing with evolutionary analyses to disentangle mechanisms of pathogen transmission.

## Methods

### Dataset construction, cluster identification and definition of DCCs

Whole genome sequencing of two collections of isolates from Manchester, UK, and the Netherlands was carried out as previously described^2^. Briefly, DNA was extracted from colony sweeps of subcultured samples before to paired-end sequencing using the Illumina HiSeq platform. These samples were combined with whole genome sequencing samples from previous studies^2,10,12–16,18,43–46^ to give a final dataset containing 2,045 samples from 1,178 patients. Samples were genotyped to the subspecies level using Mash^47^, comparing against one reference genome from each subspecies. Sample were assigned to the subspecies with the smallest genetic distance; all samples exhibited a clear subspecies assignment. A summary of all samples used, including sample accession numbers, is provided in Supplementary Table 3.

Sequencing reads from each sample were mapped against the corresponding subspecies reference sequence using the multiple_mappings_to_bam pipeline (https://github.com/sanger-pathogens/bact-gen-scripts) with BWA-MEM as the aligner. ATCC19977 (accession no. CU458896.1) was used as the reference for *M. a. abscessus* and CIP_108297 (accession no. GCF_001792625.1) for *M. a. massiliense*. SNPs were called from this alignment using the multiple_mappings_to_bam pipeline. Subspecies phylogenetic trees were reconstructed using RAxML v.8.2.12 (ref. ^48^) with the general time reversible (GTR) model of nucleotide substitution and gamma rate heterogeneity with four gamma classes. To enable extraction of maximal genetic variation, clusters of samples were identified in the subspecies trees using FastBAPS^49^. This clustering identified 19 clusters in *M. a. abscessus* and 17 clusters in *M. a. massiliense* (Extended Data Fig. 1). A summary of each FastBAPS cluster is provided in Supplementary Table 1.

All subsequent analyses were carried out on each FastBAPS cluster independently. De novo assembly was carried out on each sample as previously described^50^ and the best assembly was identified for each FastBAPS cluster on the basis of number of contigs and N50-N90 values (Supplementary Table 1). Samples from each FastBAPS cluster were mapped against their respective best assembly as above to maximize the captured SNP diversity. Recombination was removed from FastBAPS cluster alignments using Gubbins v.2.4.1 (ref. ^51^) and phylogenetic trees were reconstructed for each FastBAPS cluster as above. Phylogenetic trees were viewed and figures constructed using FigTree^52^ and GGTREE^53^.

DCCs were identified as clusters of highly related sequences collected from at least 20 patients on multiple continents. DCCs 1, 2, 3, 5 and 7 are FastBAPS clusters while DCCs 4 and 6 are subclusters within a FastBAPS cluster (Supplementary Table 1). No DCCs were identified in *M. a. bolletii*.

### Phylogenetic analyses, temporal, spatiotemporal and population reconstruction

**Extended Data Fig. 7:**
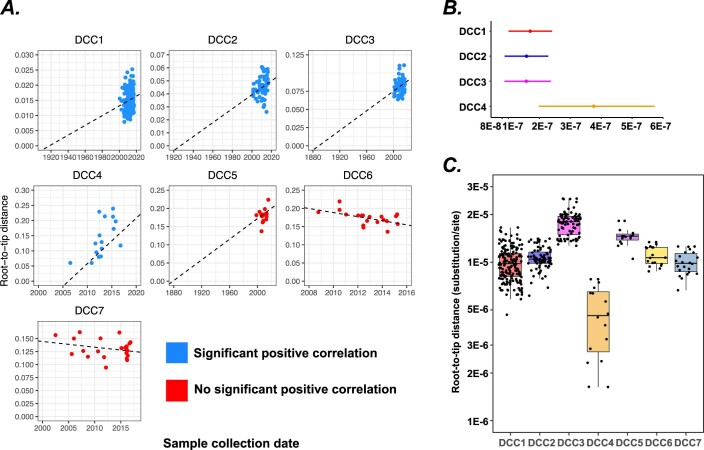
Assessment of DCC root-to-tip correlation and substitution rate comparison. (**a**) DCC root-to-tip distance vs collection date. Dashed lines represent the regression line between root-to-tip distance and collection date. Correlations were calculated from maximum likelihood phylogenetic trees. DCCs 1-4 exhibited a significant correlation based on bootstrap analyses while DCCs 5-7 did not. (**b**) Comparison of DCC 1-4 substitution rates. Substitution rate estimates are plotted for each DCC based on the posterior distribution (number of samples from the posterior distribution: DCC1 = 2255, DCC2 = 2255, DCC3 = 2255, DCC4 = 2703). Points represent median estimates and bars the 95% HPD. The substitution rate is highly similar for DCCs 1-3. (**c**) Comparison of divergence levels in each DCC. Phylogenetic trees were reconstructed for each DCC and midpoint rooted. Plotted is the root-to-tip distance (substitutions/genome site from the root of the tree each tip) for each sequence in each DCC (number of sequences: DCC1 = 172, DCC2 = 78, DCC3 = 82, DCC4 = 16, DCC5 = 15, DCC6 = 19, DCC7 = 22). DCC4 has accumulated substantially less divergence than the other DCCs. Source data

Temporal phylogenetic reconstruction was carried out on DCCs 1–7. DCC-specific datasets were constructed containing the earliest sequenced sample from each patient that clusters within the DCC. These samples were mapped against the respective DCC reference sequence (Supplementary Table 1) as above and a maximum likelihood phylogenetic tree reconstructed with RAxML^48^ as above. Methods to infer substitution rates and ancestral dates are only valid if there is a temporal signal within the dataset^54^. We initially assessed temporal signal within each dataset using root-to-tip randomization. In each case, the maximum likelihood tree was rooted to minimize the heuristic residual mean square score using TempEst^54^. We examined the root-to-tip correlation visually (Extended Data Fig. 7a) and through comparison of the *R*^2^ correlation between sample collection date and root-to-tip distance with 1,000 randomizations of the tip dates to identify significance of the correlation. A significant positive correlation was observed for DCCs 1–4 (*P* < 0.001). We therefore initially reconstructed the temporal history of these DCCs using BEAST v.2.4.2 (ref. ^19^). We used the Hasegawa–Kishono–Yano (HKY) model of nucleotide substitution. We used the relaxed log-normal clock model with a log-normal prior on the substitution rate with mean set to the estimated slope in TempEst and standard deviation 0.5. We modelled the population history using the coalescent Bayesian skyline population prior. At least three independent runs were carried out for 100,000,000 steps for each dataset. Convergence was assessed using Tracer v.1.7 (ref. ^55^).

**Extended Data Fig. 8:**
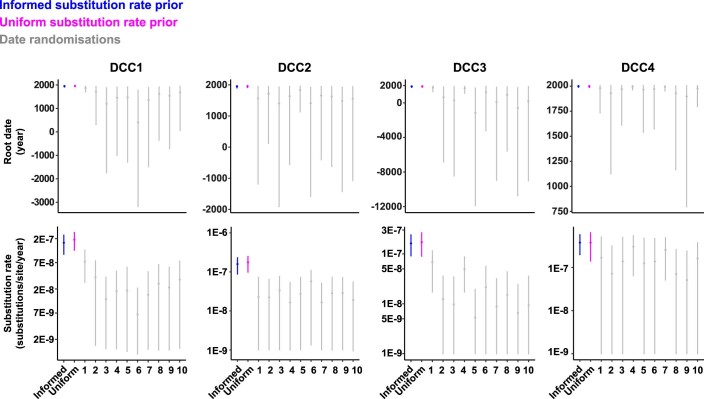
DCCs 1-4 exhibit a significant temporal signal. Date randomization test for DCCs 1-4. Points show median values and error bars the 95% HPD (number of samples from the posterior distribution: DCC1 informed, DCC1 uniform, DCC2 informed, DCC3 informed, DCC3 uniform = 2255 each, DCC4 informed, DCC4 uniform, all date randomisations = 2703 each, DCC2 uniform = 4506). The temporal evolutionary history of each DCC was reconstructed with an informed prior on the substitution rate based on root-to-tip analysis and a uniform prior (see methods). The results from these priors matched closely in all cases. We compared the tree root date and substitution rate inferences from these priors with ten date randomization runs where the sample collection dates were randomly assigned to sequences and the temporal history reconstructed. In all cases, the posterior distributions inferred with the real dates were highly different from those inferred with the date randomizations, suggesting the existence of a significant temporal signal within each DCC. Source data

As a more thorough test of temporal signal within each DCC, we carried out the date randomization test^56^. BEAST v.2.4.2 was run on each DCC dataset using a uniform substitution rate prior between 1 × 10^−9^ and 1 × 10^−5^, with these bounds chosen to encompass the likely substitution rates for *Mycobacteria* based on previous work^2,56^. Other priors were as described above. The results from these uniform prior runs were highly similar to those with the informed substitution rate prior in each case (Extended Data Fig. 8). Ten date randomizations were performed where the sequence collection dates were randomly assigned to tips. BEAST was run on each of these randomized datasets independently using the same uniform substitution rate prior (1 × 10^−9^ – 1 × 10^−5^). All four DCCs passed the date randomization test, defined here as the median posterior substitution rate and most recent common ancestor dates with the real sample collection dates not overlapping with that of any of the ten date randomizations (Extended Data Fig. 8). We did not attempt these analyses with DCCs 5–7 as they did not pass the correlation test.

The inferred substitution rates of DCCs 1–3 were highly similar (Extended Data Fig. 7b). The substitution rate of DCC4 is higher (Extended Data Fig. 7b), probably due to this clade having a far more recent common ancestor date than DCCs 1–3 (Fig. 1c) and therefore less opportunity to remove deleterious substitutions. As DCCs 5–7 contain similar levels of diversity to DCCs 1–3 (Extended Data Fig. 7c), we reconstructed their temporal history as above but using a uniform substitution rate prior with boundaries of 8.76 × 10^−^^8^ – 2.41 × 10^−^^7^, chosen as the upper and lower 95% highest probability density (HPD) substitution rate estimates for DCCs 1–3 (Extended Data Fig. 7b).

**Extended Data Fig. 9:**
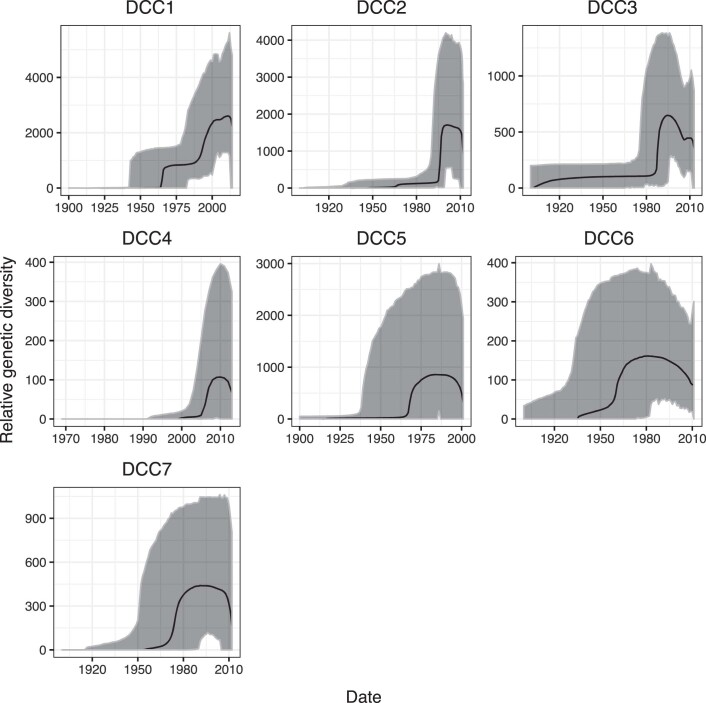
DCC Bayesian skyline plots. The relative genetic diversity (Neτ) is shown through time for each DCC as estimated from BEAST analyses. The black line represents the median estimate and the shaded area the 95% HPD. Source data

We determined whether each DCC has undergone a historical population expansion by using the Bayesian skyline plot estimates (Extended Data Fig. 9) of relative genetic diversity in the posterior distribution. We used all samples in the posterior distribution and found that all samples in all DCCs exhibited an increase in relative genetic diversity of more than tenfold relative to the value at the root of the tree, thereby strongly supporting a historical population expansion in each case. We identified the date of the expansion in each DCC by calculating the earliest date at which the relative genetic diversity increased by more than tenfold relative to the root of the tree and combined these values into a single distribution, from which the median and 95% HPD was calculated in each case.

Before carrying out spatiotemporal reconstruction, we calculated the association index^57^ of the distribution of collection continents across the maximum likelihood tree of each DCC. This was significant in each case on the basis of 1,000 location randomizations (*P* < 0.001 in each DCC), indicating a correlation between phylogeny and continent of collection. We carried out asymmetric phylogeographic reconstructions of DCCs 1–3 using the BEAST_CLASSIC package v.1.3.2 in BEAST v.2.4.2 (ref. ^19^). Each sequence was labelled with the continent of collection. We used an informed log-normal substitution rate prior and Bayesian skyline population prior as above. We used an exponential prior on the overall rate of lineage movements with mean 1.0. The relative rates of migration between different continent pairs were modelled using a gamma distribution with alpha and beta both set to 1.0. As the number of sequences collected from each continent is unequal for each DCC, we assessed the robustness of our inferences by randomly subsampling the sequences from overrepresented continents and rerunning the spatiotemporal reconstruction. We carried out the subsampling five times and found that the results were highly similar in all subsamples (for example, Fig. 2b). Supported migration routes were identified using SPREAD v.0.9.6 (ref. ^58^) as directed continent pairs had Bayes factor support greater than three in the dataset without subsampling and at least four of the five subsampled datasets.

### Mutational spectrum analysis

**Extended Data Fig. 10:**
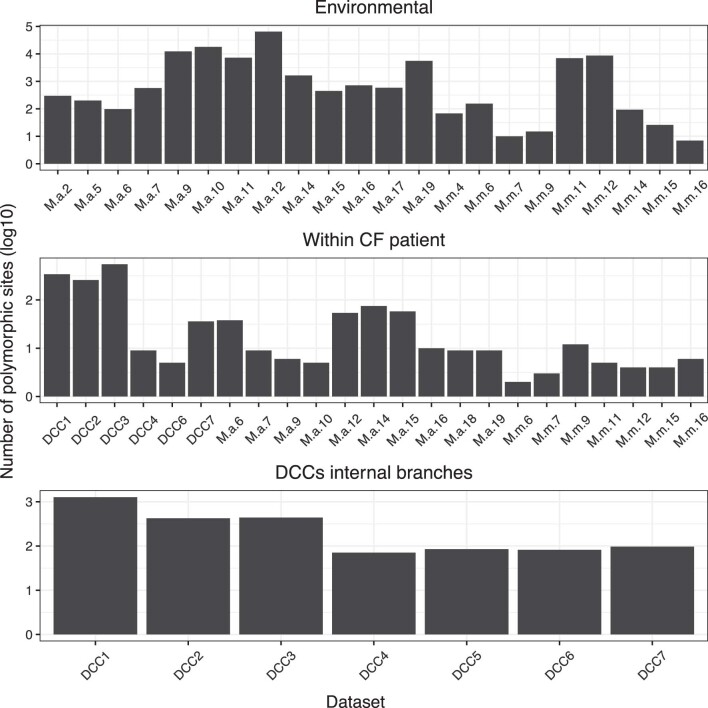
Polymorphic sites used in the mutational spectra analyses. The number of alignment sites within each FastBAPS cluster used for mutational spectrum analysis of the environmental, within-CF patient and DCC internal branches is shown. Some of these sites will have mutated on multiple occasions within a FastBAPS cluster so the number of polymorphic sites is not the same as the number of mutations. M.a: *M. abscessus subsp abscessus*. M.m: *M. abscessus subsp massiliense*. Source data

The mutational spectrum consists of all of the mutations that have occurred within the history of a sample set in their surrounding nucleotide context^31^. It is necessary to identify the direction of each mutation, that is, the parental nucleotide and the descendent nucleotide. To identify the mutations that have occurred and their direction, we carried out ancestral reconstruction on each FastBAPS cluster phylogenetic tree. Recombination was removed and phylogenetic trees reconstructed as above. Ancestral reconstruction was carried out on all variable alignment positions using the phylogenetic analysis by maximum likelihood (PAML) package v.4.9 (ref. ^59^). We compared the fit of HKY, HKY + GAMMA, GTR and GTR + GAMMA models of nucleotide substitution. Results were highly similar with all models and in all cases either the GTR or GTR + GAMMA model was supported. The mutations that occurred along each branch in the phylogeny were extracted from the PAML output. The surrounding nucleotide context of each mutation was identified from the reference sequence that was mapped against. The number of polymorphic sites contributing to each mutational spectrum is shown in Extended Data Fig. 10.

To compare the mutational spectrum in different niches, the branches in the phylogenetic tree were divided into categories (Fig. 3a). We reasoned that the internal branches within the non-DCC clusters will have been environmental as these branches probably often span hundreds to thousands of years during which time prolonged human infection will have been unlikely. We therefore calculated the environmental mutational spectrum by combining contextual mutations inferred along all internal branches of non-DCC clusters.

The phylogenetic branches within clades containing sequences from a single patient represent within-patient evolution. We therefore calculated the within-patient mutational spectrum by combining contextual mutations inferred along branches within monophyletic patient clades.

The relative contributions of environmental and within-patient evolution along tip branches and branches leading to patient ancestors is unclear as the patient may have acquired the infection at any point along this branch. We therefore did not include these branches in the environmental mutational spectrum or the within-patient spectrum.

To examine the mutations acquired during DCC transmission chains, we combined the contextual mutations that occurred along the internal branches within the seven DCC trees (Fig. 3a). We excluded the deep branches in these clades that precede population expansion (Fig. 3a) to only examine mutations that have occurred since emergence and therefore in more recent transmission chains.

We compared mutational spectra between niches by subtracting the inferred environmental spectrum from the DCC internal branch and within CF patient spectra. Significance of observed differences was assessed through 1,000 independent down-samplings of the inferred environmental mutations to the number identified along DCC internal branches or within CF patients. Contextual mutations were identified as significant if their calculated proportion fell outside two standard deviations of the mean proportion in the 1,000 replicates. This process was repeated ten times and all reported significant mutations were significant in all ten runs.

Decomposition of the mutational spectrum into input signatures was carried out using signal^32^ (https://signal.mutationalsignatures.com/, date last accessed 15 November 2020). The contextual mutations that were elevated in the DCCs relative to the environment were combined into a 10,000 mutation catalogue with their relative frequencies representing their relative enrichment above the environment. This catalogue was used as input for signal specifying lung as the originating organ. Mutational drivers were assigned from the respective COSMIC mutational signature (https://cancer.sanger.ac.uk/cosmic/signatures/SBS/index.tt, date last accessed 15 November, 2020).

### Transmission network reconstruction

SNP distances were calculated between all pairs of samples within each FastBAPS cluster using PairSNP^60^ and the minimum SNP distance between each pair of patients extracted. Patients were classified as being linked at a given SNP cut-off if their closest pair of samples differed by that number of SNPs or fewer. Localized linkages were classified on the basis of available metadata if the patients were in the sample hospital, CF Trust, city or state.

Transmission networks were reconstructed on the basis of minimum SNP distance between patient isolates. SNP distances of 20 and 38 SNPs were previously shown to represent ‘probable’ and ‘possible’ transmission, respectively, on the basis of the number of SNPs observed in within patient infections^2^. We therefore plotted the transmission network at ten SNPs to represent very likely transmission and 38 SNPs to represent possible transmission.

Transmission network connectivity measures (Extended Data Fig. 6) were calculated using 38 SNPs as a cut-off to include linkages, with 38 chosen to include possible person-to-person transmission events^2^. Therefore, any patient linkages of 39 SNPs or more were not included. The total number of linkages involving patients with or without CF was identified and divided by the total number of patients within the respective group to calculate the average number of transmission linkages per patient with and without CF. To calculate the weighted connectivity measures, each edge in the transmission network was given a weighting of a 39-SNP distance. Therefore, edges linking patient pairs whose isolates differ by zero SNPs were given a weighting of 39 and edges had zero weighting if they connect patient pairs whose isolates differ by 39 or more SNPs. The total weighting of all edges involving patients with or without CF was identified and divided by the total number of patients within the respective group to calculate the average weighted connectivity. To identify the average weighting of CF–CF, CF–non-CF and non-CF–non-CF linkages, the total weighted connectivity of each edge type was calculated by summing the weights of all respective edges and this was divided by the total number of potential linkages of that type.

### Trends in tobacco smoking

Annual estimates of the number of cigarette sales per adult per day were obtained from https://ourworldindata.org/smoking (last accessed 18 March 2021). 30 countries were included with data available from before 1960.

### Reporting Summary

Further information on research design is available in the Nature Research Reporting Summary linked to this article.

## Supplementary information

Reporting Summary

Supplementary Tables Supplementary Tables 1–3.

## Data Availability

All source data files are available at 10.5281/zenodo.5116229. Accession numbers of all samples used in analyses are included in this source data and in Supplementary Table 3. Source data are provided with this paper.

## Source data

Source Data Fig. 1 Phylogenetic trees and population expansion distributions for Fig. 1.
Source Data Fig. 2 Phylogeographic trees and migration distributions for Fig. 2.
Source Data Fig. 3 Mutational spectra and signature proportions for Fig. 3.
Source Data Fig. 4 SNP distances and associated metadata for Fig. 4.
Source Data Extended Data Fig. 1 Phylogenetic trees for Extended Data Fig. 1.
Source Data Extended Data Fig. 2 Population expansion distributions, CF life expectancy and smoking data for Extended Data Fig. 2.
Source Data Extended Data Fig. 3 Clade ages and descendent continents for Extended Data Fig. 3.
Source Data Extended Data Fig. 4 Phylogenetic trees and associated metadata for Extended Data Fig. 4.
Source Data Extended Data Fig. 5 SNP distances for Extended Data Fig. 5.
Source Data Extended Data Fig. 6 SNP distances for Extended Data Fig. 6.
Source Data Extended Data Fig. 7 Root-to-tip distances and substitution rates for Extended Data Fig. 7.
Source Data Extended Data Fig. 8 Ancestor data and substitution rate distributions for Extended Data Fig. 8.
Source Data Extended Data Fig. 9 DCC Bayesian skyline plots for Extended Data Fig. 9.
Source Data Extended Data Fig. 10 Number of polymorphic sites in each dataset for Extended Data Fig. 10.

## Code availability

Code used for data analysis is available at 10.5281/zenodo.5116229.
