# Supplementary material for: Dissemination of Mycobacterium abscessus via global transmission networks
Source: Nat Microbiol. 2021 Sep 20;6(10):1279–88. doi: 10.1038/s41564-021-00963-3 (PMC8478660; doi:10.1038/s41564-021-00963-3)
Supplement: Supplementary file 1 — Reporting Summary [file 41564_2021_963_MOESM1_ESM.pdf]

nature research

Corresponding author(s): R. Andres Floto, Julian Parkhill

Last updated by author(s): Jul 20, 2021

Reporting Summary

Nature Research wishes to improve the reproducibility of the work that we publish. This form provides structure for consistency and transparency in reporting. For further information on Nature Research policies, see our Editorial Policies and the Editorial Policy Checklist.

Statistics

For all statistical analyses, confirm that the following items are present in the figure legend, table legend, main text, or Methods section.

n/a	Confirmed	
☐	☒	The exact sample size (*n*) for each experimental group/condition, given as a discrete number and unit of measurement
☐	☒	A statement on whether measurements were taken from distinct samples or whether the same sample was measured repeatedly
☐	☒	The statistical test(s) used AND whether they are one- or two-sided<br>*Only common tests should be described solely by name; describe more complex techniques in the Methods section.*
☒	☐	A description of all covariates tested
☒	☐	A description of any assumptions or corrections, such as tests of normality and adjustment for multiple comparisons
☐	☒	A full description of the statistical parameters including central tendency (e.g. means) or other basic estimates (e.g. regression coefficient) AND variation (e.g. standard deviation) or associated estimates of uncertainty (e.g. confidence intervals)
☐	☒	For null hypothesis testing, the test statistic (e.g. *F*, *t*, *r*) with confidence intervals, effect sizes, degrees of freedom and *P* value noted<br>*Give P values as exact values whenever suitable.*
☐	☒	For Bayesian analysis, information on the choice of priors and Markov chain Monte Carlo settings
☒	☐	For hierarchical and complex designs, identification of the appropriate level for tests and full reporting of outcomes
☒	☐	Estimates of effect sizes (e.g. Cohen's *d*, Pearson's *r*), indicating how they were calculated

Our web collection on statistics for biologists contains articles on many of the points above.

Software and code

Policy information about availability of computer code

Data collection	No software was used in collection of the data
Data analysis	Phylodynamic and phylogeographic analyses were performed with BEAST v2.4.2. Convergence of log files was examined with Tracer v1.7. Examination of temporal signal was carried out with TempEst v1.5.1. Read mapping was performed using the multiple_mappings_to_bam pipeline v1.6. Cluster assignment was carried out using FastBAPS v1.0.0. Initial assignment of samples to subspecies was carried out using mash v2.1.1. Recombination was removed using gubbins v2.4.1. Reconstruction of non-temporal trees was carried out with RAxML v8.2.12. Trees were viewed with FigTree v1.4.4 and GGTREE v3. Supported inter-continental movements were identified with SPREAD v0.9.6. Nucleotide substitutions were reconstructed onto phylogenetic trees using PAML v4.9. SNP distances between sequence pairs were calculated using PairSNP v0.0.1. Additional custom scripts, including those used to calculate mutational spectra are available in GitHub at DOI https://doi.org/10.5281/zenodo.4279139.

For manuscripts utilizing custom algorithms or software that are central to the research but not yet described in published literature, software must be made available to editors and reviewers. We strongly encourage code deposition in a community repository (e.g. GitHub). See the Nature Research guidelines for submitting code & software for further information.

Data

Policy information about availability of data

All manuscripts must include a data availability statement. This statement should provide the following information, where applicable:

- Accession codes, unique identifiers, or web links for publicly available datasets
- A list of figures that have associated raw data
- A description of any restrictions on data availability

The accession codes for all sequences used in this study are provided in Table S3. All source data, including alignments, phylogenetic trees, BEAST XML files and mutational spectra have been deposited in https://doi.org/10.5281/zenodo.5116229.

Field-specific reporting

Please select the one below that is the best fit for your research. If you are not sure, read the appropriate sections before making your selection.

☐ Life sciences ☐ Behavioural & social sciences ☒ Ecological, evolutionary & environmental sciences

For a reference copy of the document with all sections, see nature.com/documents/nr-reporting-summary-flat.pdf

Ecological, evolutionary & environmental sciences study design

All studies must disclose on these points even when the disclosure is negative.

Study description	Phylogenetic clustering of available whole genome sequences followed by phylodynamic and phylogeographic analysis of selected clones. Mutational spectrum analysis comparing between clusters. Transmission network analysis of whole genome sequences.
Research sample	Raw data was 2045 whole genome sequencing samples of Mycobacterium abscessus available on the European Nucleotide Archive. This data represents the global population structure of Mycobacterium abscessus
Sampling strategy	We included all available whole genome sequencing samples which capture the diversity from over 1100 patients on five continents
Data collection	Data was downloaded from the European Nucleotide Archive, sample metadata was obtained from authors of previous studies or data tables in previous studies
Timing and spatial scale	We used all available while genome sequencing samples, which were collected between 1998 and 2017. These samples were collected from 16 countries on 5 continents. We retained all available samples to maximise sequence, temporal and spatial diversity
Data exclusions	No data were excluded from the study
Reproducibility	All Bayesian analyses involved at least three independent runs and results were highly similar between runs. Phylogeographic analyses were carried out with all data and with five independent down-samplings of over-represented locations. All repeats were successful and all repeat analyses showed the same results
Randomization	Samples were divided into clusters based on phylogenetic relationships. Cluster cut-offs were assigned using hierarchical Bayesian analysis of population structure
Blinding	All samples were coded by unique lab identification and therefore all associated metadata are anonymised

Did the study involve field work? ☐ Yes ☒ No

Reporting for specific materials, systems and methods

We require information from authors about some types of materials, experimental systems and methods used in many studies. Here, indicate whether each material, system or method listed is relevant to your study. If you are not sure if a list item applies to your research, read the appropriate section before selecting a response.

Materials & experimental systems

n/a	Involved in the study
☒	☐ Antibodies
☒	☐ Eukaryotic cell lines
☒	☐ Palaeontology and archaeology
☒	☐ Animals and other organisms
☒	☐ Human research participants
☒	☐ Clinical data
☒	☐ Dual use research of concern

Methods

n/a	Involved in the study
☒	☐ ChIP-seq
☒	☐ Flow cytometry
☒	☐ MRI-based neuroimaging
